# Comparative analysis of mouse bone marrow and adipose tissue mesenchymal stem cells for critical limb ischemia cell therapy

**DOI:** 10.1186/s13287-020-02110-x

**Published:** 2021-01-13

**Authors:** Pegah Nammian, Seyedeh-Leili Asadi-Yousefabad, Sajad Daneshi, Mohammad Hasan Sheikhha, Seyed Mohammad Bagher Tabei, Vahid Razban

**Affiliations:** 1 Department of Molecular Medicine, School of Advanced Medical Sciences and Technologies, Shiraz University of Medical Sciences, Shiraz, Iran; 2 Department of Molecular Medicine, School of Medicine, Shahid Sadoughi University of Medical Sciences, Yazd, Iran; 3 Postdoctoral Researcher, Stem Cells Technology Research Center, Shiraz University of Medical Sciences, Shiraz, Iran; 4 Biotechnology Research Center, International Campus, Shahid Sadoughi University of MedicalSciences, Yazd, Iran; 5 Research and Clinical Center for Infertility, Shahid Sadoughi University of Medical Sciences, Yazd, Iran; 6 Department of Genetics, Shiraz University of Medical Science, Shiraz, Iran; 7 Maternal-fetal Medicine Research Center, Shiraz University of Medical Sciences, Shiraz, Iran; 8 Stem Cells Technology Research center, Shiraz University of Medical Sciences, Shiraz, Iran

**Keywords:** Mesenchymal stem cells, Angiogenesis, Cell therapy, Critical limb ischemia

## Abstract

**Introduction:**

Critical limb ischemia (CLI) is the most advanced form of peripheral arterial disease (PAD) characterized by ischemic rest pain and non-healing ulcers. Currently, the standard therapy for CLI is the surgical reconstruction and endovascular therapy or limb amputation for patients with no treatment options. Neovasculogenesis induced by mesenchymal stem cells (MSCs) therapy is a promising approach to improve CLI. Owing to their angiogenic and immunomodulatory potential, MSCs are perfect candidates for the treatment of CLI. The purpose of this study was to determine and compare the in vitro and in vivo effects of allogeneic bone marrow mesenchymal stem cells (BM-MSCs) and adipose tissue mesenchymal stem cells (AT-MSCs) on CLI treatment.

**Methods:**

For the first step, BM-MSCs and AT-MSCs were isolated and characterized for the characteristic MSC phenotypes. Then, femoral artery ligation and total excision of the femoral artery were performed on C57BL/6 mice to create a CLI model. The cells were evaluated for their in vitro and in vivo biological characteristics for CLI cell therapy. In order to determine these characteristics, the following tests were performed: morphology, flow cytometry, differentiation to osteocyte and adipocyte, wound healing assay, and behavioral tests including Tarlov, Ischemia, Modified ischemia, Function and the grade of limb necrosis scores, donor cell survival assay, and histological analysis.

**Results:**

Our cellular and functional tests indicated that during 28 days after cell transplantation, BM-MSCs had a great effect on endothelial cell migration, muscle restructure, functional improvements, and neovascularization in ischemic tissues compared with AT-MSCs and control groups.

**Conclusions:**

Allogeneic BM-MSC transplantation resulted in a more effective recovery from critical limb ischemia compared to AT-MSCs transplantation. In fact, BM-MSC transplantation could be considered as a promising therapy for diseases with insufficient angiogenesis including hindlimb ischemia.

## Introduction

Critical limb ischemia (CLI) is the most severe form of peripheral artery disease (PAD) in which associates with cardiovascular events, ulceration, high risk of limb amputation, infection, gangrene, and death [1, 2]. CLI causes by atherosclerosis that leads to narrowing and obstructions of arteries that supply the lower extremities [3]. In addition, CLI shows the appearance of extensive atherosclerosis related to smoking, diabetes, hypercholesterolemia, and age [4]. Twenty percent of the mortality rate during 6 months after the diagnosis and 50% at 5 years reported. The cause of this extensive mortality rate probably relates to the systemic aspect of cardiovascular diseases [5]. Standard therapy for increasing blood flow to the affected extremity is surgical or endovascular revascularization and limb amputation [6]. While revascularization is the first-line treatment for CLI, medical therapy also considers a required therapeutic supplement. The main purpose of medical therapy is to inhibit myocardial infarction, stroke, and death, but it further helps to speed up wound healing, impede amputation, and also improve quality of life [7]. However, nearly half of patients with CLI are not suitable for revascularization procedures because of high operative risk or undesirable endovascular anatomy [8]. As a result, the use of experimental stem cell therapy (SCT) and also gene therapy in order to stimulate angiogenesis emerged as a promising alternative for the treatment of disorders related to limb ischemia [9, 10].

### Background on stem cell therapy for CLI

In the last few years, investigators have used cellular therapies in order to improve blood flow to the ischemic limb via transplanting cells that have the potential to induce the formation of new blood vessels [11]. As a matter of fact, cell-based therapies have been explored as a promising treatment for CLI patients who have no option for surgical or endovascular revascularization [11, 12]. Different cell types have been used including bone marrow-derived mononuclear cells (BMMNCs), bone marrow mesenchymal stem cells (BMMSCs), endothelial progenitor cells (EPCs), blood-derived progenitor cells, adipose tissue mesenchymal stem cells (ATMSCs), and other bone marrow-derived progenitors [13, 14]. In fact, mesenchymal stem cell (MSC) transplantation has been proposed as a novel and effective treatment approach for tissue engineering and regenerative medicine for diverse disease states like ischemic disorders, including CLI [15, 16].

MSCs are multipotent non-hematopoietic and fibroblast-like adherent cells that can isolate from different tissue sources, such as the bone marrow, placenta, adipose tissue, dental pulp, and umbilical cord blood [17, 18]. They are able to differentiate into various types of cells such as the bone, cartilage, fat, and muscle and also show specific surface antigen expression. In addition to the ability of cell differentiation, MSCs perform their therapeutic effects through the secretion of paracrine factors that have angiogenic, anti-apoptotic, anti-inflammatory, and immunomodulatory effects [19]. As a matter of fact, they can promote reparative processes via secretion of soluble molecules, MSC-derived growth factors, and extracellular matrix components that have paracrine mechanisms and also differentiation into specific cells [20–22].

Investigations about stem cell-based therapy for CLI focus on the use of bone marrow mesenchymal stem cells and support by clinical evidences [23]. Mesenchymal stem cells (MSCs) consist of nearly 0.001 to 0.08% of cells within the bone marrow and are nonhematopoietic stromal cells be able to differentiate into mesenchymal lineages, such as the muscle, bone, cartilage, and fat. They can be culture-expanded easily to harvest a large number of cells. In addition, these cells can be stored for both autologous and allogeneic use [24]. In numerous preclinical studies, the therapeutic angiogenesis of bone marrow mesenchymal stem cells (BMMSCs) was proved, and also, there are strong evidences that BMMSCs have the potential to produce growth factors, angiogenesis-related cytokines, chemokines and extracellular matrix molecules, and also have similar cellular and molecular properties with pericytes [25, 26]. Several animal experiments demonstrated that transplanted BMMSCs could induce more advantages than BMMNCs for the treatment of CLI [27]. The intramuscular (IM) injection of MSCs in animals with CLI indicated zonal angiogenesis and improved blood flow via paracrine support of injured cells and a process of local progenitor cell differentiation, and also the regulation of angiogenic factors secretion. Furthermore, MSCs involved in the restoration of vascular integrity and neovascularization after injury via direct differentiation into blood vessel cells and also upregulate proangiogenic factors expression [28, 29].

Therapeutic application of stem cells for the treatment of CLI does not have to be derived only from the bone marrow but also MSCs from the adipose tissue can be used [30]. In the last few years, the adipose tissue used a lot in tissue repair and regeneration owing to its variety of ingredients and functions [31]. The benefits of adipose tissue cells are the abundant source, stable accessibility, simple procedures of collection, and also possess nearly 40 times more stem cells than the bone marrow [32]. Adipose tissue-derived stem cells (ASCs) indicate stable growth and proliferation kinetics and can differentiate into chondrogenic, osteogenic, myogenic, adipogenic, and neurogenic lineages. In addition, the differentiation potential of ASCs into vascular smooth muscle cells and vascular endothelial cells (VECs) was demonstrated [33]. ASCs secrete angiogenesis-related cytokines, such as vascular endothelial growth factor (VEGF) and hepatocyte growth factor (HGF), in order to induce angiogenesis in ischemic tissue [34]. Wang et al. investigated the therapeutic effect of ASCs on the infarcted rat hearts, and their results indicated that ASCs can improve cardiac function, hinder ventricular remodeling, and increase angiogenesis in the infarct border zone after MI [35]. The application of autologous ASCs in a mouse model of ischemic limb disease indicates that the transplantation of ASCs leads to a fast improvement of blood flow and enhances capillary density in the ischemic muscle tissue [36]. Fan et al. examined the distribution and kinetics of engrafted ASCs after transplantation in a mouse hindlimb ischemia model via 3D multimodality imaging technique and realized that ASCs perform proangiogenic effects through a VEGF/mechanistic target of rapamycin/Akt-dependent pathway [37]. Moreover, the angiogenic potential of xenogeneic (human) ASCs assessed in a mouse model of hindlimb ischemia, and the transplantation of hASCs has shown to induce restoration from ischemic muscle injury and to enhance capillary density through paracrine secretion of angiogenic molecules [38].

## Materials and methods

### Isolation and culture of mouse BM-MSCs

**Fig. 1:**
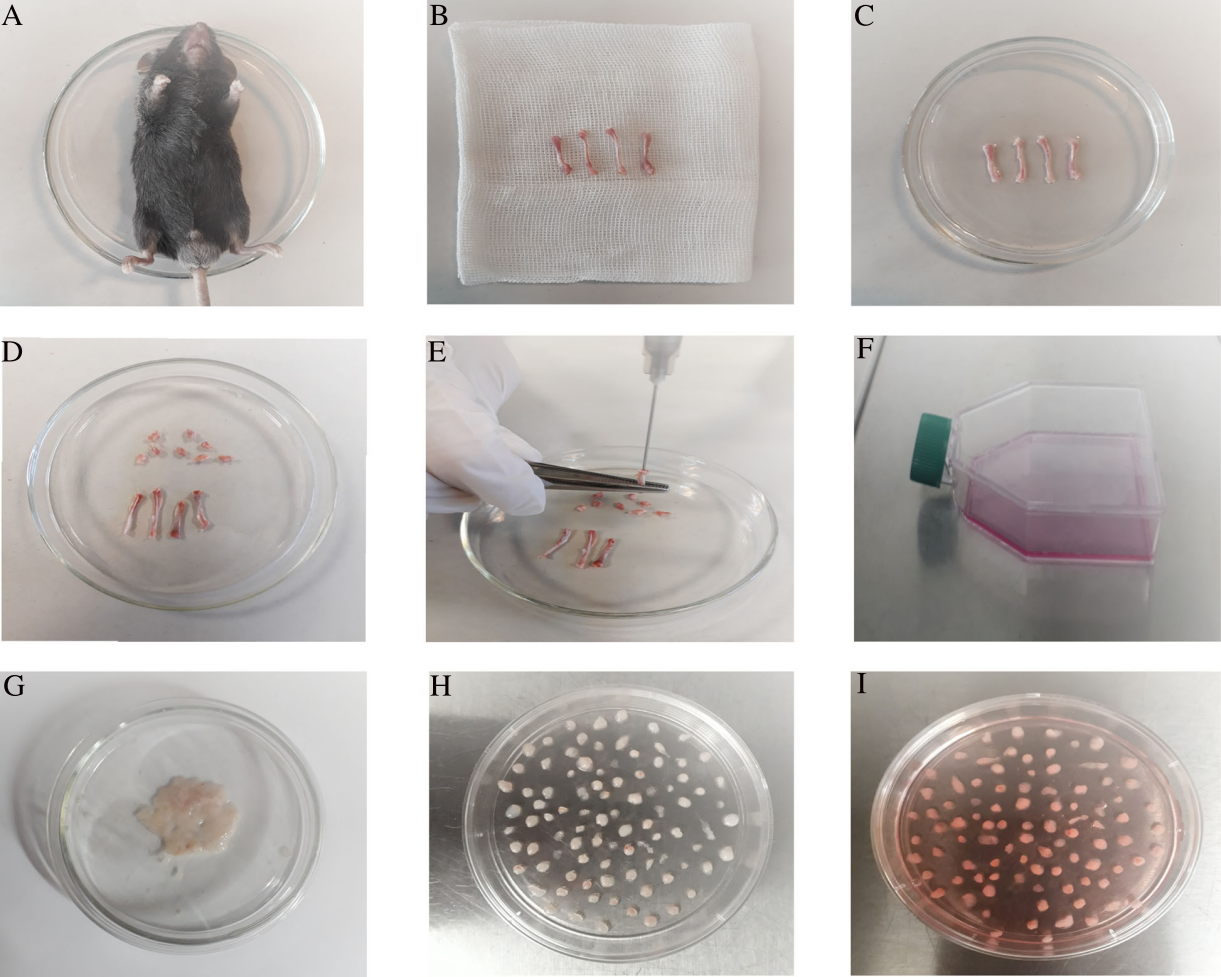
Images of the bone marrow- and adipose-derived mesenchymal stem cell collection procedures. **a** First, the mouse was sacrificed by cervical dislocation. **b** The tibia and femur bones were dissected, and then, the muscles, ligaments, and tendons were removed. **c** The bones were transferred to a 100-mm sterile culture dish with 5 mL phosphate-buffered saline containing penicillin–streptomycin. **d** The dish was transferred into the biosafety cabinet and the two ends of the bones below the end of the marrow cavity were cut with scalpel. **e** A 5-ml syringe was inserted into the bone cavity and used to slowly flush the marrow out. The bone cavities were washed until the bones became pale. **f** Finally, all the bone pieces were removed and the media transferred to a new flask that incubated at 37 °C in a 5% CO_2_ incubator. **g** For adipose-derived MSCs, the inguinal adipose tissue was harvested from sacrificed mice and washed with sterile phosphate-buffered saline to remove contaminating debris. **h** The adipose tissue were minced into 1–2 mm^3^ pieces using sterile scalpel and place the tissue explants in culture dish around 5 mm space between adjacent fragments. **i** Finally, growth medium was added gently to the Petri dish and incubated at 37 °C in a 5% CO_2_ incubator

To isolate the bone marrow, 6-week-old C57BL/6 mice were killed by cervical dislocation, then the ends of the tibia and femur bones were cut to expose the marrow. A 5-ml syringe containing complete media used to extract the cells via flushing the marrow plug out of the cut end of the bone with 1 ml of complete media and collect in a 15-ml tube. Strong flushing is necessary during marrow cell preparation. Then, the cell pellet derived from 2 tibia and 2 femur bones was suspended in a growth medium containing Dulbecco’s modified Eagle’s medium (DMEM) high glucose (Gibco, USA) and 10% fetal bovine serum (FBS; Gibco, USA) with 100 U/mL penicillin–streptomycin (Gibco, USA) and cultured in a 75-cm^2^ culture flask and were maintained at 37 °C and 5% CO_2_. Nonadherent cells were removed after 24 h, and the flask was washed with phosphate-buffered saline (PBS; Gibco, USA). The medium was changed regularly every 3–4 days, and at approximately 70% confluence, the cells were detached using trypsin-EDTA (Gibco, USA) and transferred to new flasks; these cells were considered as passage 1 (Fig. 1a–f).

### Isolation and culture of mouse AT-MSCs

Inguinal adipose tissue was harvested from 6-week-old C57BL/6 mice and washed with sterile phosphate-buffered saline (PBS; Gibco, USA) to remove contaminating debris. The adipose tissue was minced into 1–2 mm^3^ pieces using a sterile scalpel and placed in the tissue plate 70 mm under sterile conditions. The tissue explants were placed in a culture dish around 5 mm space between adjacent fragments. After adhesion of explants to the plate surface (10 min), growth medium containing Dulbecco’s modified Eagle’s medium (DMEM) high glucose (Gibco, USA) and 20% fetal bovine serum (FBS; Gibco, USA) with 100 U/mL penicillin–streptomycin (Gibco, USA) was added gently to the Petri dish without disturbing the explants. After 48 h, the medium was replaced with fresh medium. On reaching 80–90% confluence, the cells were subcultured after treatment with 0.05% trypsin-EDTA (Gibco, USA). The cells were incubated in a 37 °C humidified incubator with 5% CO2 and 95% air. At passage one, the culture medium was changed to DMEM containing 10% FBS. All of the culture medium replaced every 3–4 days (Fig. 1g–i).

### Evaluation of MSC differentiation

#### Osteogenesis

For differentiation to osteocyte, passage 3 BM-MSCs and passage 3 AT-MSCs were incubated to differentiate into osteoblasts in corresponding induction medium for 2 weeks. The cells were maintained in control and osteogenic media. The control medium consisted of DMEM (Gibco, USA), supplemented with 10% FBS (Gibco, USA) and 1% penicillin/streptomycin (Gibco, USA). The osteogenic medium contained DMEM, 15% FBS (Gibco, USA), 100 μM l-ascorbic acid, 10 mM glycerol 3-phosphate, and 100 nM dexamethasone (Sigma-Aldrich). The medium was replaced 2 times a week. After 2 weeks the cultured cells were stained with Alizarin Red Solution to visualize calcium deposits. The cells were fixed in 4% paraformaldehyde (PFA) for 20 min and stained using Alizarin Red Solution for 20 min at room temperature.

#### Adipogenesis

For adipocyte differentiation, passage 3 BM-MSCs and passage 3 AT-MSCs were incubated to differentiate into adipocytes in corresponding to the induction medium for 2 weeks. The cells were maintained in control and adipogenic media. The control medium consisted of DMEM (Gibco, USA), supplemented with 10% FBS (Gibco, USA) and 1%penicillin/streptomycin (Gibco, USA). The adipogenic medium contained DMEM, 15% FBS, 100 μM L-ascorbic acid, 200 μM Indomethacin, 1000 nM insulin (Sigma-Aldrich), and 100 nM dexamethasone (Sigma-Aldrich). The medium was replaced 2 times a week. After 2 weeks, the cultured cells were stained with Oil Red-O Solution to visualize lipid vacuoles. The cells were fixed in 4% paraformaldehyde (PFA) for 20 min and stained using Oil Red-O Solution for 20 min at room temperature.

### Phenotypic characterization

For the analysis of surface marker expression of BMMSCs and ATMSCs, flow cytometry was performed. The expression of surface markers was evaluated using monoclonal antibodies against mouse anti-CD44 FITC, anti-CD105 perCP, anti-CD45 FITC, and anti-CD34 PE. Both types of cells at passage 4 were washed with PBS and then harvested via incubation in 0.05% trypsin/EDTA. After centrifugation, the cell pellets were washed with PBS and resuspended in 1 ml PBS. Then, the cells were stained with the specific antibodies in darkness. After incubation for 30 min at 4 °C, the cells were washed with PBS and then analyzed by flow cytometry. The data was analyzed with Flow Jo software (version 7.6).

### Wound healing assay (scratch assay)

Wound-healing assay on endothelial cells, human umbilical vein endothelial cell (HUVEC) (purchased from pasteur institute of iran), was used to study the paracrine effect of BM-MSCs and AT-MSCs on endothelial cell migration potential. After 24-h FBS starvation of MSCs, the supernatants from cells were collected. The wound-healing assay was employed by scratching the monolayer HUVEC confluent cell cultures using a sterile plastic micropipette tip. The cells were washed by DMEM and PBS twice for smoothing the edges of the scratch and removing floating cells. After that, wound healing initiated by adding the collected supernatants from MSCs on HUVEC cultures in different wells of a 6-well plate. After incubating the cells at 37 °C in 5% CO_2_ for 0, 15, 18, 20, 22, and 24 h, the migration images of the cells were observed under a microscope with ×10 (Nikon, ECLIPSE, TS100), the distance of cell migration was measured, and images were taken using ImageJ Analysis software.

### Hindlimb ischemia model and cell transplantation

All animal experiments were performed according to the guidelines approved by the Shiraz University of Medical Sciences ethics committee (1397.430).

Male C57BL/6 mice, weighing 25–30 g (6–8 week-old) were purchased from the Comparative and Experimental Medicine center at Shiraz University of Medical Sciences and were selected randomly. Animals were kept in standard conditions (12 h light and 12 h darkness, temperature of 18–22 °C, and 55 ± 5% humidity). They were fed a standard mouse diet and given water ad libitum.

For creating critical limb ischemia (CLI) model, all animals in each group were anesthetized intraperitoneal (IP) administration using ketamine 10% (100 mg/kg, Alfasan CO., Netherlands) and xylazine 2% (5 mg/kg, Alfasan CO., Netherlands). Animals were placed in dorsal recumbency. A skin incision (about 10 mm) was done along the center of the medial thigh from the abdomen towards the knee. Subcutaneous fat tissue superficial was gently pushed away to expose the femoral neurovascular bundle. The femoral artery pushed away from the femoral vein and nerve. Subsequently, the femoral artery was isolated by blunt dissection from the femoral vein at the ligation sites between the proximal caudal femoral artery and the bifurcation of the deep femoral artery and saphenous artery. Then, two sutures were passed using 6–0 silk, transected and cauterized. The incision was closed using continuous 5–0 vicryl sutures. The operation was done under a surgical microscope (Zeiss OP-MI6 SD; Carl Zeiss, Goettingen, Germany).

The male mice were randomly divided into the following three groups (*n*  =  12/group): (1) control group: CLI was performed and received PBS into the ischemic site; (2) BM-MSC group: CLI was performed and received BM-MSCs; and (3) AT-MSC group: CLI was performed and received AT-MSCs. To transfer the cells into the ischemic leg, intramuscularly (IM) injection of 5 × 10^5^ BM-MSCs and AT-MSCs that resuspended in PBS as treatment groups and PBS as a control group was done at four different sites of gastrocnemius (GC) muscle after 24 h.

### Functional scoring: evaluation of the recovery of the damaged limbs after MSC transplantation

Semi-quantitative assessments of limb function and ischemia were performed using the Tarlov, ischemia, modified ischemia, function, and the grade of limb necrosis scoring system at days 3, 7, 14, 21, and 28 after cell transplantation. The degree of ischemic damage was evaluated through indicators, such as skin color changes, swelling, and grade of the limb necrosis. On the other hand, mice were examined preoperatively, immediately postoperatively but after recovery from anesthesia, and at day 3, and at 1, 2, 3, and 4 weeks after induction of hind limb ischemia (Table 1).

**Table 1:** Functional scales

Tarlov score	Description
0	No movement
1	Barely perceptible movement, non–weight bearing
2	Frequent movement, non–weight bearing
3	Supports weight, partial weight bearing
4	Walks with mild deficit
5	Normal but slow walking
6	Full and fast walking
Ischemia score	Description
0	Autoamputation > half lower limb
1	Gangrenous tissue > half foot
2	Gangrenous tissue < half foot, with lower limb muscle necrosis
3	Gangrenous tissue < half foot, without lower limb muscle necrosis
4	Pale foot or gait abnormalities
5	Normal
Modified ischemia score	Description
0	Autoamputation of leg
1	Leg necrosis
2	Foot necrosis
3	Discoloration of 2 toes
4	Discoloration of 1 toe
5	Discoloration of > 2 nails
6	Discoloration of 1 nail
7	No necrosis
The grade of limb necrosis	Description
0	Normal limb without swelling, necrosis or atrophy of muscle
1	Necrosis limiting to toes (toes loss)
2	Necrosis extending to a dorsum pedis (foot loss)
3	Necrosis extending to a crus (knee loss)
4	Necrosis extending to a thigh (total hind-limb loss)
Function score	Description
0	Dragging
1	No plantar flexion
2	No toe flexion
3	No grabbing force
4	Some grabbing force
5	Normal

### Assessment of donor cell survival

To identify the presence of grafted male cells in the female GC muscle, the Y chromosome-specific SRY gene was assessed by a polymerase chain reaction. At days 3, 7, 14, 21, and 28 after treatment, the GC muscle was collected, genomic DNA was extracted using Trizol (Sigma-Aldrich), and then, SRY analysis was performed. The forward primer sequence was TTTATGGTGTGGTCCCGTGGTGAG, and the reverse primer sequence was TTGGAGTACAGGTGTGCAGCTCTAC.

### Immunohistochemical staining and histological analysis

Histological analysis of the limb tissues was done at days 7, 14, 21, and 28 after cell transplantation. To confirm that the target cells induced angiogenesis in the gastrocnemius muscle, immunohistologic staining was performed with an anti-CD31 antibody (Biocare Medical), a marker for vascular endothelial cells. The mice were euthanized at predetermined times, and gastrocnemius was removed and fixed in 10% formalin. After fixation, the tissue was embedded in paraffin, and the tissue sections were prepared and mounted on slides. Then, the tissue section slides were stained with hematoxylin-eosin (H&E) and assessed by microscopy (Olympus BX43, Shinjuku, Japan). The tissue samples were deparaffinized by xylene and ethanol, and then, antigen retrieval was performed with proteinase K treatment. After this treatment, the tissue samples were incubated with an anti-CD31 monoclonal primary antibody at 100 μL for 15 min at room temperature. After being washed with TBS, the samples were incubated with a master polymer plus HRP at room temperature for 30 min. Then, samples were washed with TBS. Chromogen solution was prepared, 1 drop of DAB Chromogen concentrate to 1 ml of DAB substrate buffer. Samples were treated with DAB and incubated at room temperature for 5 min. The samples were covered with hematoxylin for 15 s and then washed with distilled water. After that, the dehydration step was performed by alcohol solutions at room temperature for 30 s. Finally, the samples were cleared in xylene and mounted with a permanent mounting medium. Tissue sections were analyzed using the vascular hotspot technique to obtain MVD. Sections were scanned at low power to determine areas of the highest vascular density. Within this region, individual microvessels were counted in three separate random fields at high power (0.142 mm^2^ field size). The mean vessel count from the three fields was used. A single countable microvessel was defined as any endothelial cell or group of cells that were clearly separated from other vessels, stroma, or tumor cells without the necessity of a vessel lumen or RBC within the lumen. Areas of gross hemorrhage and necrosis were avoided. MVD counts were measured by counting CD31 IHC hot spots in three separate 400× fields.

### Statistical analysis

Statistical analysis was performed using GraphPad Prism 7 Software. Results are expressed as the mean standard error of the mean. Comparisons between multiple groups were performed using one-way analysis of variance (ANOVA), with post hoc testing performed with Bonferroni analysis or unpaired *t* tests, as appropriate. *P* values ≤ 0.05 were considered statistically significant (*p < .05, **p < .01, ***p < .001, ****p < .0001). Data were presented as mean ± standard deviation (SD).

## Results

### Characterization of mouse BM-MSCs and AT-MSCs

**Fig. 2:**
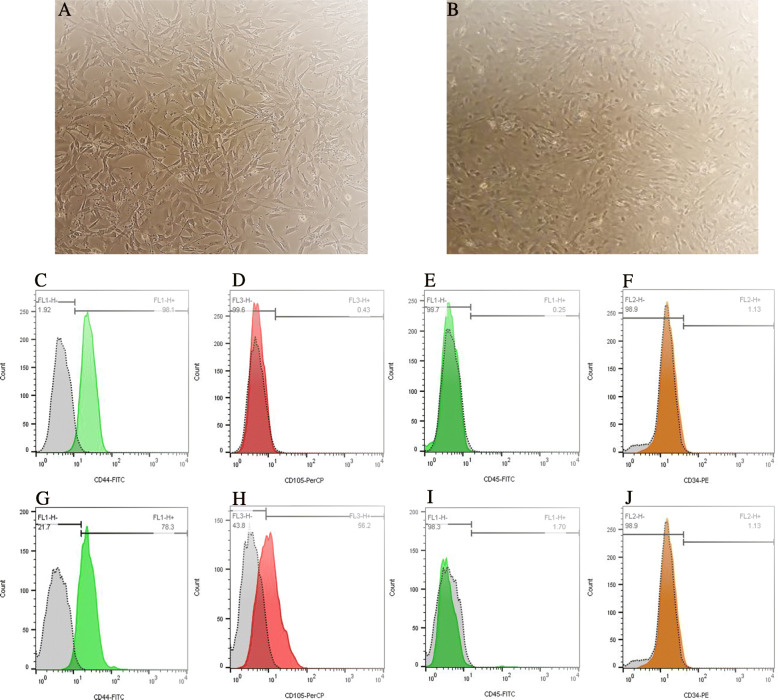
Morphology of the bone marrow (**a**) and adipose mesenchymal stem cells (**b**) under inverted microscope (10×) and cell surface markers of C57BL/6 mice bone marrow and adipose mesenchymal stem cells. Flow cytometry analysis results showed that BM-MSCs were positive for MSC marker CD44 (**c**) and negative for CD105 (**d**). Cells were negative for hematopoietic cell marker CD45 (**e**) and CD34 (**f**). AT-MSCs were positive for MSC markers CD44 (**g**) and CD105 (**h**) and negative for hematopoietic markers CD45 (**i**) and CD34 (**j**)

Figure 2a, b represents the spindle-shaped morphology of BM-MSCs and AT-MSCs at passage 3 that obtained from the bone marrow and adipose tissue of C57BL/6 mice. To characterize the phenotypes of BM-MSCs and AT-MSCs, flow cytometry was performed to analyze the surface markers of MSCs. Cells labeled with FITC, perCP, and PE-conjugated antibodies and examined by flow cytometry. Cells are dissociated and stained with CD34, CD45 (hematopoietic cell markers) and CD44, CD105 (mesenchymal stem-cell markers). Results showed that the BM-MSCs were strongly positive for MSC marker CD44 and negative for cell markers CD105, CD34, and CD45 (Fig. 2c–f). And AT-MSCs were positive for MSC markers CD44 and CD105 and negative for cell markers CD34 and CD45 (Fig. 2g–j).

### In vitro differentiation capacities of mouse BM-MSCs and AT-MSCs

**Fig. 3:**
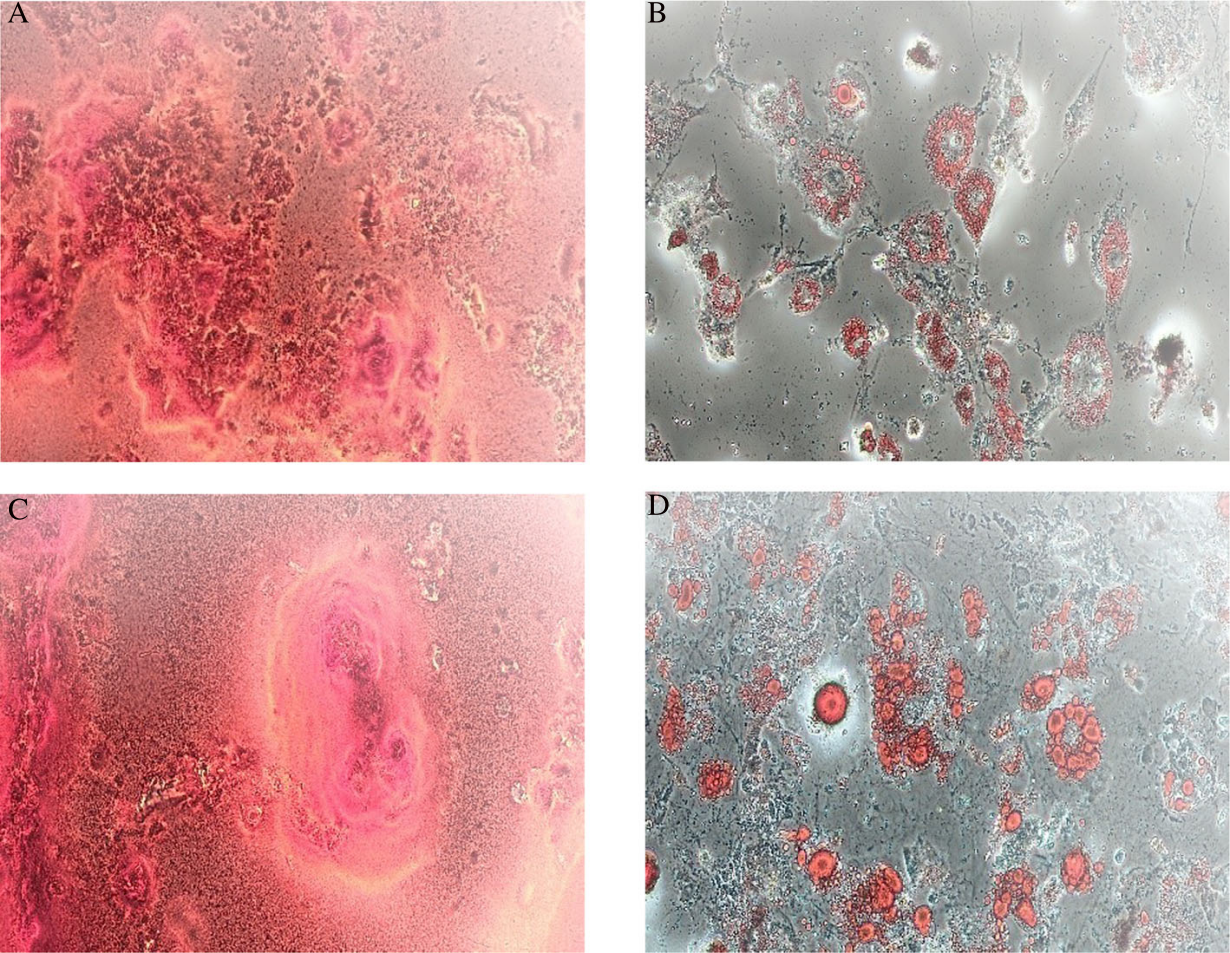
Differentiation capacity of the mouse bone marrow and adipose mesenchymal stem cells, BM-MSCs and AT-MACs. For BM-MSCs, osteogenesis differentiation demonstrated by calcium deposition in Alizarin red staining (**a**) and adipogenesis differentiation demonstrated by vacuoles containing fat in Oil Red staining (**b**). For AT-MACs, the cells differentiated into osteocytes, stained with alizarin red (**c**), and adipocytes stained with oil red (**d**); ×10 magnification

The differentiation capacities of BM-MSCs and AT-MSCs to osteocyte and adipocyte obtained using Alizarin Red staining and Oil Red-O staining, respectively. For BM-MSCs, Alizarin Red staining showed that mineralized nodules formed in the BM-MSCs after 2 weeks under the osteogenic induction. Also Oil Red-O staining demonstrated that lipid-rich vacuoles formed after 2 weeks under the adipogenic induction (Fig. 3a, b). For AT-MSCs, Alizarin red staining demonstrated that mineralized nodules formed after 2 weeks under the osteogenic induction and also intracellular Oil-red-O staining showed lipid-rich vacuoles formed after 2 weeks under the adipogenic induction (Fig. 3c, d).

### Capacity of BM-MSCs and AT-MSC supernatant in cell migration

**Fig. 4:**
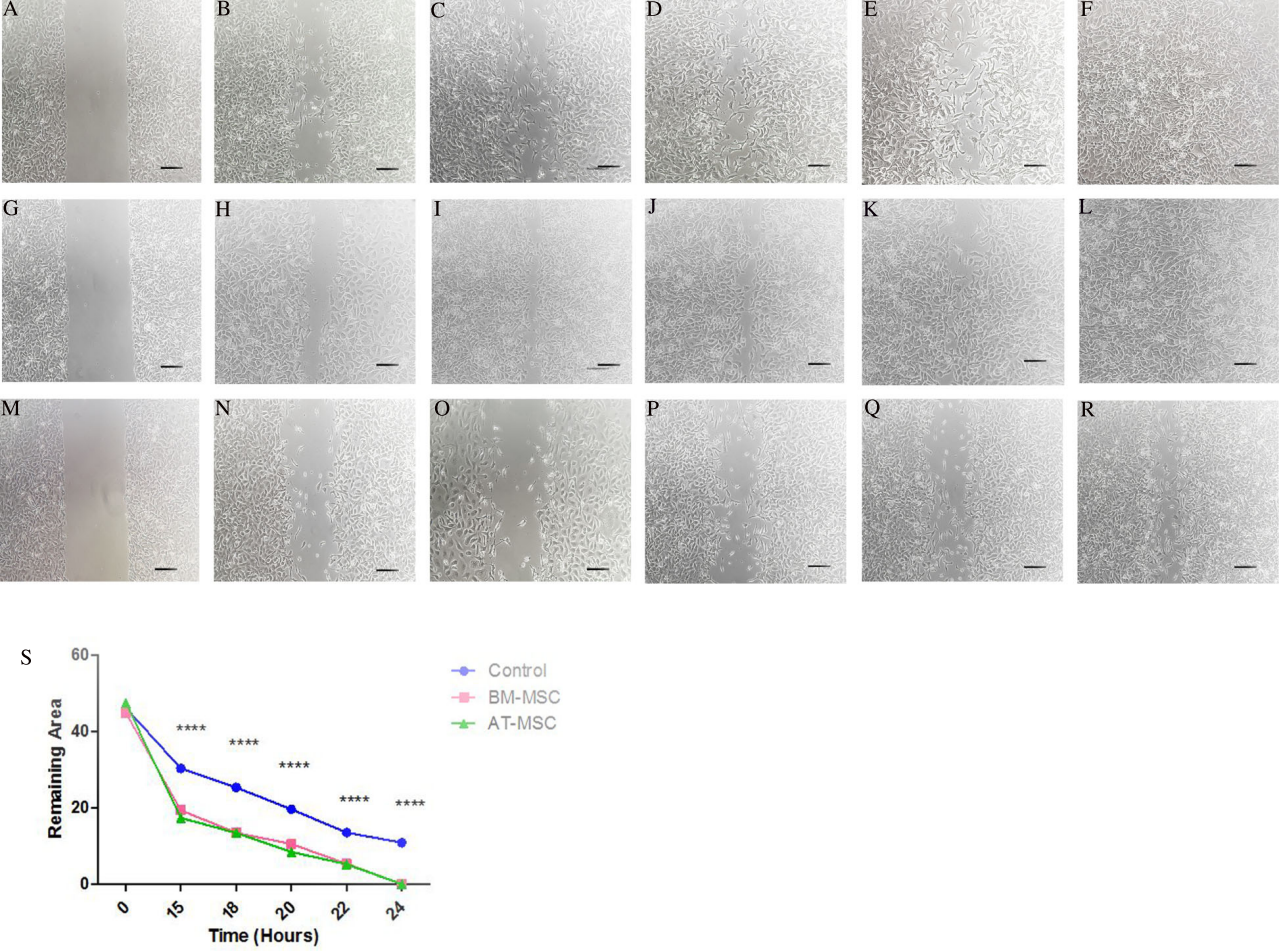
For BM-MSC-conditioned medium, the images in a time series (T0, T15, T18, T20, T22, T24) were analyzed for gap area over time (**a**–**f**). For AT-MSC-conditioned medium, the images in the same time series were analyzed (**g**–**l**). Photomicrographs from the control group in a defined time series (**m**–**r**). Graphical comparison of the mean ingrowth and standard deviation (SD) of control, BM-MSC, and AT-MSC groups. *P* values ≤ 0.05 were considered statistically significant (s); ×10 magnification, scale bar 200 μm

Scratch assay was performed on the target cells. The results showed that BM and AT-MSCs supernatant led to increased migration potential of endothelial cells (HUVECs) through a paracrine manner. The supernatant of both cells led to the complete closure of the wound after 24 h. At time points 15, 18, 20, 22, and 24 h post-scratch, we documented a significant percentage decrease in the scratch area remaining between the BM-MSC compared to the control group (19.44 ± 0.61, 13.5 ± 0.33, 10.59 ± 0.5, 5.37 ± 0.25, 0 ± 0, respectively, *p* < 0.0001 for all time points). Additionally, at time points 15, 18, 20, 22, and 24 h post-scratch, the AT-MSC group showed a significant decrease in scratch area remaining compared to the control group (17.44 ± 0.51, 13.49 ± 0.4, 8.4 ± 0.21, 5.2 ± 0.16, 0 ± 0, respectively, *p* < 0.0001 for all time points). In fact, supernatants from both cell sources showed similarity in their ability to stimulate cell migration compared with the control group. For the control group, no scratch was 100% healed by the end of the 24-h experiment. The photos taken with a microscope in a time series then analyzed by ImageJ software in order to calculate the distance between gaps (Fig. 4).

### BM-MSCs and AT-MSC transplantation improves recovery of ischemia-induced injury to mouse hindlimbs

**Fig. 5:**
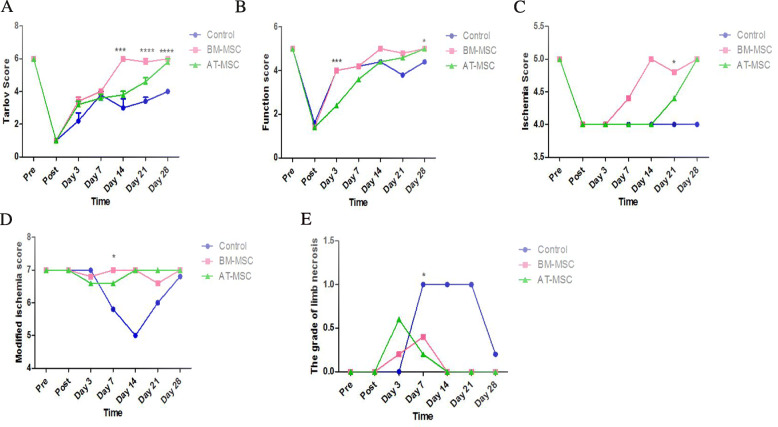
MSC treatment (5 × 10^5^) improves hind limb ischemia. BM-MSC-treated mice indicated accelerated functional recovery according to Tarlov score at days 14, 21, and 28 (*p* <  0.0001 for all time points) and Function score at days 3 and 28 (*p* = 0.0004, *p* = 0.01) (**a**, **b**). BM-MSC-treated mice had greater improvement of ischemia compared with the control group as shown by ischemia score at day 21 (*p* = 0.02), modified ischemia score at day 7 (*p* = 0.01) (**c**, **d**). BM-MSCs and AT-MSCs had similar effect on improvement of necrotic changes according to the grade of limb necrosis score at day 7 (*p* = 0.02) (**e**)

In order to test the in vivo function of BM-MSCs and AT-MSCs in ischemic hindlimb injury, the day after surgery mice were treated with PBS as control group and 5 × 10^5^ BM-MSCs and AT-MSCs injected into the gastrocnemius muscle. Muscle strength testing was performed in the gastrocnemius muscle during 28 days after treatment. The activity of the limbs was evaluated by Tarlov and Function scores, and severity of ischemic changes was evaluated by ischemia, modified ischemia, and the grade of limb necrosis scores. Our results indicated that all MSC-transplanted mice and non-treated mice moved with difficulty after the first day of transplantation; in fact, mice did not walk well and only dragged their feet. Over 28 days, BM-MSC-treated mice showed improved functional outcomes compared with AT-MSCs and control group, with accelerated improvement in the Tarlov score at days 14, 21, and 28 (6 ± 0, *p* < 0.0001, 5.8 ± 0.44, *p* < 0.0001, 6 ± 0, *p* < 0.0001; Fig. 5a) and Function score at days 3 and 28 (4 ± 0, *p* = 0.0004, 5 ± 0, *p* = 0.01; Fig. 5b). In AT-MSC-treated mice group, the process of recovery was not significant as much as BM-MSC-treated mice group, but it was much better than the control group. In fact, the rate of hindlimb recovery was significantly increased in BM-MSC and also increased in AT-MSCs transplanted mice after 28 days. On the contrary, in PBS group, there were no mice that fully recovered. The grade of ischemia improved in BM-MSC-treated mice using the ischemia score at day 21 (4.8 ± 0.44, *p* = 0.02; Fig. 5c) and the modified ischemia score at day 7 (7 ± 0, *p* = 0.01, Fig. 5d). The degree of ischemic damage was assessed through indicators, such as swelling, skin color changes, and grade of the limb necrosis. Necrotic changes were macroscopically evaluated using the grade of limb necrosis score, and in BM-MSCs and AT-MSCs treated mice groups, ischemic damage recovery was observed at day 7 (0.4 ± 0.54, *p* = 0.02, 0.2 ± 0.44, *p* = 0.02, respectively, Fig. 5e).

### PCR for SRY gene

**Fig. 6:**
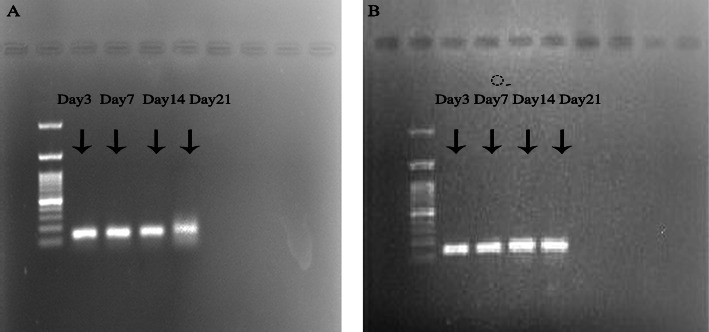
PCR for SRY gene showed that both BM (**a**) and AT-MSC cells (**b**) were survived in the GC muscle at days 3, 7, 14, and 21

Polymerase chain reaction for Y chromosome gene SRY indicated that at days 3, 7, 14, and 21, both donor male cells, BM-MSCs and AT-MSCs, were survived in the GC muscle of female mice. But at day 28, no SRY gene was detected in the GC muscle for both cells (Fig. 6).

### Evaluation of capillary density and neoangiogenesis

**Fig. 7:**
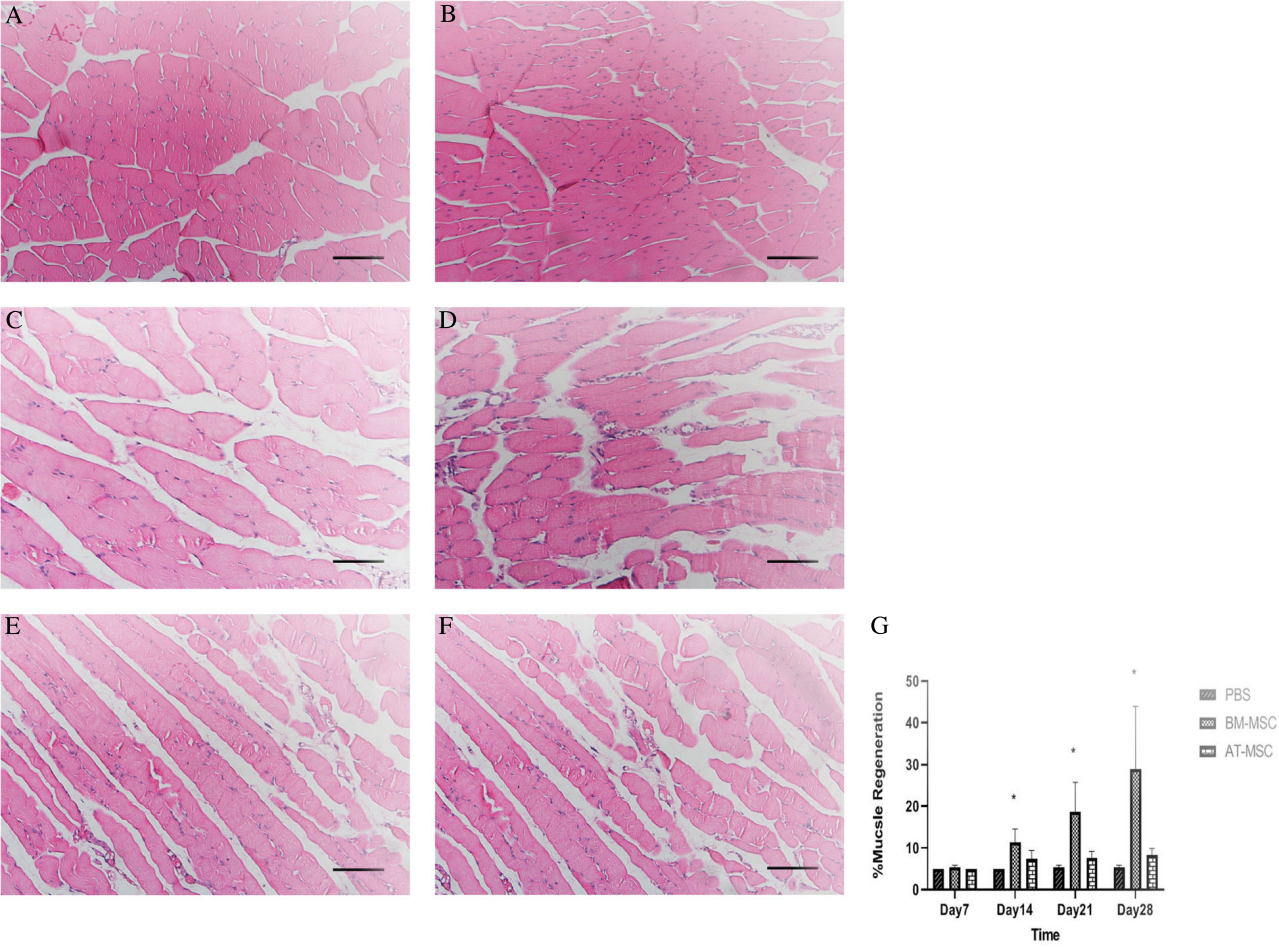
Histological analysis after H&E staining. Gastrocnemius muscle regeneration characterized by the presence of centrally located nucleus in mice treated with BM-MSCs (**a**, **b**), AT-MSCs (**c**, **d**), and control groups (**e**, **f**) at days 14 and 21. Quantification of the percentage (mean ± SD) of muscle regeneration (*p* ≤ 0.05) (**g**); ×100 magnification, scale bar, 50 μm

**Fig. 8:**
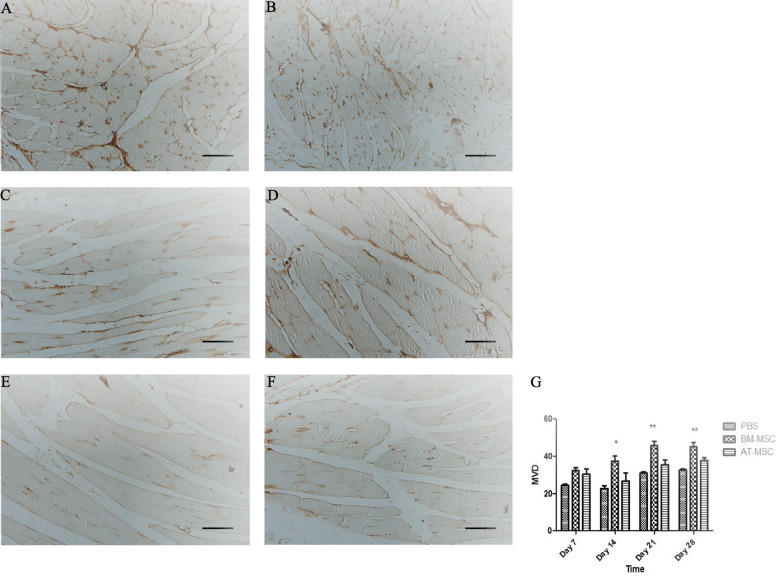
Histological analysis of the limb muscles. Immunohistochemical staining of BM-MSC (**a**, **b**), AT-MSC (**c**, **d**), and PBS (**e**, **f**) groups with anti-CD31 antibody at days 14 and 21. Quantification of capillary density. Capillary density was counted after CD31 staining (*p* ≤ 0.05) (**g**). ×100 magnification, scale bar, 50 μm

The gastrocnemius muscles were collected at days 7, 14, 21, and 28 after cell transplantation. In order to understand the angiogenic effect of BM-MSCs and AT-MSCs in the ischemic muscles at the cellular level, the gastrocnemius muscles were stained with H&E and the percentage of muscle regeneration was evaluated; in addition, capillary density was assessed morphometrically by examining three fields per sections after immunofluorescence staining for endothelial cells with an anti-CD31 antibody. The analysis of muscle regeneration in gastrocnemius muscle indicated that the muscles from BM-MSC-treated mice had a large increase in muscle regeneration compared with AT-MSCs and control groups starting from day 14. BM-MSC-treated mice showed increased gastrocnemius muscle regeneration from 5% at day 7 up to 40% at day 28. In fact, BM-MSCs had greater percentage in muscle regeneration at days 14, 21, and 28 compared with AT-MSCs and control groups (11.33 ± 3.2, *p* = 0.02, 18.66 ± 7.09, *p* = 0.05, 29 ± 14.93, *p* = 0.04, respectively) (Fig. 7). Results from IHC showed increased vasculature in BM-MSC-treated mice at days 14, 21, and 28 compared with AT-MSCs and control group (37.33 ± 4.6, *p* = 0.03, 45.66 ± 4, *p* = 0.005, 45 ± 4.3, *p* = 0.006, respectively). In fact, CD31-MVD in BM-MSC-treated mice was significantly higher than the other groups. CD31-MVD did not significantly differ between AT-MSCs and control groups (26.66 ± 7.6, 35.33 ± 4.5, 37.66 ± 2.5, *p* value > 0.05 for all time points, 22.66 ± 2.5, 31 ± 1, 32 ± 1.1, *p* value > 0.05 for all time points) (Fig. 8).

## Discussion

Ischemia is a condition related to damage of the blood vessels which thereby damage cells via depriving them of oxygen and nutrients. Peripheral arterial disease like CLI ischemia results in a serious pathological conditions that affect different organs. Therapies for CLI have limited efficacy, but in recent years, therapies have been developed to treat this disease like stem cell therapy. Recent clinical studies suggest that stem cell transplantation is a reliable therapeutic option in selected patients with CLI [39, 40]. In this study, we focus on the therapeutic neovascularization effect of BM-MSCs and AT-MSCs and provide insights into their potential for clinical use as a cell-based therapy for CLI. Sustained angiogenesis is a crucial process and play an important role in improvements of CLI. For the first step, we successfully isolated BM-MSCs from the bone marrow and AT-MSCs from the adipose tissue. The cells were then assessed for the characteristic MSC phenotypes including MSC CD markers and differentiation capacity. There are three criteria to define MSCs; in fact, MSCs should exhibit some of these properties. First, MSC must show fibroblast-like shape. Second, expression of CD44, CD73, and CD90 and negative for CD14, CD34, and CD45. Third, the cells must be able to differentiate to osteoblasts, adipocytes, and chondroblasts [41]. Both cells expressed MSC phenotypic markers. These typical MSC markers included CD34, CD45, CD44, and CD105. BM-MSCs were strongly positive for MSC marker CD44 and negative for cell markers CD105, CD34, and CD45, and AT-MSCs were positive for MSC markers CD44 and CD105 and negative for cell markers CD34 and CD45. Our results indicated that both BM-MSCs and AT-MSCs successfully differentiated into osteoblasts and adipocytes. After 2 weeks, the cells showed calcium depositions and oil droplets in the cytoplasm and stained positive with alizarin red and oil red dye, respectively. These data demonstrated that both BM-MSCs and AT-MSCs exhibited the characteristic MSC phenotypes. In order to investigate the ability of MSCs on endothelial cell migration and neovascularization, scratch assay was performed on the HUVEC cells. In this study, we showed that 24 h after scratch initiation both BM-MSCs and AT-MSC supernatant resulted in a significant percentage decrease in the scratch area remaining compared to the control group. BM-MSCs compared to AT-MSCs resulted in a faster closure of the wound, although this difference was not so great. In fact both cells possess powerful paracrine effect on endothelial cell proliferation and migration. It is well known that MSCs perform their paracrine effects via secrete soluble factors which are angiogenic, anti-apoptotic, anti-inflammatory, and immunomodulatory. MSCs can secrete significant amounts of growth factors and cytokines that can promote new vessel formation and remodeling of injured tissues such as proangiogenic factors VEGF, bFGF, MMPs, IL-8, and HGF. On the other hand, one of the most important property of MSCs is their capacity for promoting angiogenesis and proangiogenic factor VEGF is the main growth factor for this process that secreted by MSCs during pathophysiological conditions [42, 43]. The BM-MSCs and AT-MSCs were then assessed for their ability to treat critical limb ischemia. We used a model of limb ischemia that was induced by the complete removal of the femoral artery and the closing of its branches. CLI-induced mice were evaluated for functional recovery and ischemia using the Tarlov, ischemia, modified ischemia, function, and the grade of limb necrosis scoring system at days 3, 7, 14, 21, and 28 after cell transplantation [44, 45]. The results showed that BM-MSC transplantation improved the treatment efficacy of hindlimb ischemia compared to AT-MSCs and control groups. We observed remarkable improvements in function and foot mobility with BM-MSC treatment according to Tarlov and Function scores. In fact, muscle function and muscle strength, which are a very important parameter to evaluate the angiogenic effects of MSCs therapy, improved during 28 days after BM-MSC treatment. Additionally, the mice showed restructuring of the skeletal muscles at the injured sites and functional improvements. Mice in the BM-MSC group improved necrosis of the hindlimb and also showed reduced swelling and edema. In fact, according to our observational tests results, BM-MSCs compared to AT-MSCs resulted in a faster and more effective recovery from limb ischemia because of its therapeutic properties. Our results showed similarity to several pre-clinical trials. These approaches indicated that BM-MSC transplantation significantly improves functional recovery of CLI model through induction of angiogenesis [46, 47]. To evaluate angiogenesis at the cellular level, H&E and immunohistochemical staining were done for the evaluation of muscle regeneration and detection of CD31 endothelial cells marker. Assessment of neovascularization and capillary density was done by MVD score [48, 49]. Our results from H&E and IHC confirmed the results obtained from the behavioral tests. Muscle regeneration in BM-MSC group had a significant increase compared to AT-MSC group during 28 days, starting from day 14. On the other hand, it confirmed that neovascularization and blood supply were increased at the GC muscle. IHC results indicated that formation of the new blood vessels at day 7 was not significantly differ in 3 groups, but at days 14, 21, and 28, neovascularization in BM-MSC-treated mice was greater than AT-MSCs and control groups. The potential of BM-MSCs in regeneration of the new blood vessels was confirmed by MVD score. According to our results, MVD in the BM-MSC group was increased after day 7 compared with two other groups. This property of BM-MSCs in promoting angiogenesis is related to the vascular differentiation capacity of these cells as well as secretion of angiogenic growth factors and cytokines [50]. In fact, these results showed that histologic findings were associated with vascular and functional outcomes. On the other hand, according to our results from behavioral, H&E, and IHC tests, we observed improvements in muscle function and muscle strength with BM-MSC treatment as well as formation of the new blood vessels and muscle regeneration. Additionally, AT-MSC-treated mice showed improvements in functional recovery, but it was not effective as much as BM-MSC group. BM-MSCs can contribute to angiogenesis via secretion of VEGF, angiogenin, IL-8, HGF, TNF-alpha, PD-ECGF, FGF-2, and MMP-9. VEGF plays a critical role in angiogenesis through stimulating endothelial cell proliferation, migration, and organization into tubules. Furthermore, VEGF can increase the number of circulating endothelial progenitor cells [51–53]. At day 28, after the treatment, the presence of donor cell was assessed by PCR for SRY gene. The results showed that both BM and AT-MSCs were survived during 21 days at the GC muscle. These results indicated important information concerning immunologic response after allogeneic MSC transplantation to the GC muscle. One of the most important limitations of MSC transplantation to treat limb ischemia is poor survival of the transplanted cells because of adverse microenvironment in injured site such as hypoxia, ischemia, and also excessive inflammation [54, 55]. Previous studies demonstrated that BM-MSCs secrete various cytokines that have immunomodulatory, anti-inflammatory, and anti-apoptotic functions in the target position of the transplantation such as IL-10, IL-6, PGE2, and TGF-β [56, 57]. Some strategies are to reinforce the longevity of transplanted cells such as ways of delivery, number of injected cells, preconditioning of cells, genetic modification, and combined cell therapy [58]. In this study, we used IM injection as a delivery way and defined number of cells, and according to the results, these methods were effective in cell survival. It should be noted that the number of cells reduce during 28 days because of unfavorable microenvironment, and at the end of the period, few of the cells remain. MSCs have been broadly studied because of their great potential for regenerative medicine [59]. BM-MSCs participate in angiogenesis to improve ischemia and restore blood flow via directly differentiating into vascular cells and secretion of proangiogenic cytokines; in fact, MSC secretome involves abundant angiogenic factors, growth factors, and cytokines [60, 61]. Paracrine proangiogenic properties have been defined to mesenchymal stem cells, both in vitro and in vivo [62, 63]. BM-MSCs secrete significant amounts of cytokines and proangiogenic factors such as VEGF, bFGF, MMPs, IL-8, and HGF which play a key role in neovascularization and promote new vessel formation and remodeling of injured tissues [64]. In angiogenesis-dependent diseases like CLI, the main purpose of therapy is to improve angiogenesis and restore blood formation. With due attention to the regeneration properties and better angiogenic potential of BM-MSCs compared to AT-MSCs, BM-MSC transplantation is growing up as an effective therapy for treatment of such diseases [65].

A limitation in our study was the lack of Laser Doppler Imaging (LDI) to evaluate and monitor blood stream into the hindlimb during the regeneration process. However, the results of our functional, molecular, and cellular tests indicated that BM-MSC transplantation compared to AT-MSCs had better therapeutic effects on critical limb ischemia treatment. The evidences from induction of endothelial cell migration, muscle functional improvements, muscle restructure, necrosis grade, muscle regeneration, and formation of new blood vessels demonstrate that BM-MSC transplantation has a great potential to treat hindlimb ischemia. On the other hand, we observed all of the above factors that associated with improvements of neovascularization and blood supply were significantly elevated in BM-MSC-treated mice compared to AT-MSCs and control groups. According to our results obtained from cellular, molecular, and behavioral tests and also from previous studies that detected the level of angiogenic factors and cytokines, BM-MSCs compared to AT-MSCs have greater angiogenic potential and also secretes more amount of angiogenic factors such as VEGF and IL-8, which confirms the importance of selection of cell type for stem cell-based regenerative therapies in order to induce angiogenesis [66, 67]. With regard to these findings, we could say that BM-MSCs could be a promising therapy to treat diseases with insufficient angiogenesis like critical limb ischemia compared to AT-MSCs.

## Conclusions

The present study demonstrated the significant role of BM-MSCs in the treatment of limb ischemia. We observed improvements in functional recovery, and according to previous studies, BM-MSCs can promote neovascularization in ischemic tissues mainly through secretion of different growth factors, proangiogenic factors, and cytokines. In conclusion, BM-MSC transplantation could be considered as a promising therapy for diseases with insufficient angiogenesis including hindlimb ischemia.

## Data Availability

All data generated or analyzed during this study are included in this published article.

## Abbreviations

CLI: Critical limb ischemia
PAD: Peripheral arterial disease
MSCs: Mesenchymal stem cells
BM-MSCs: Bone marrow mesenchymal stem cells
AT-MSC: Adipose tissue mesenchymal stem cell
SCT: Stem cell therapy
BMMNCs: Bone marrow-derived mononuclear cells
EPCs: Endothelial progenitor cells
VECs: Vascular endothelial cells
VEGF: Vascular endothelial growth factor
HGF: Hepatocyte growth factor
MI: Myocardial infection
DMEM: Dulbecco’s modified Eagle’s medium
FBS: Fetal bovine serum
PFA: Paraformaldehyde
PBS: Phosphate-buffered saline
HUVECs: Human umbilical vein endothelial cell
TGF: Transforming growth factor
